# Clinical Phenotype Classification of Atrial Fibrillation Patients Using Cluster Analysis and Associations with Trial-Adjudicated Outcomes

**DOI:** 10.3390/biomedicines9070843

**Published:** 2021-07-20

**Authors:** Marco Vitolo, Marco Proietti, Alena Shantsila, Giuseppe Boriani, Gregory Y. H. Lip

**Affiliations:** 1 Liverpool Centre for Cardiovascular Science, University of Liverpool and Liverpool Heart & Chest Hospital, Liverpool L7 8TX, UK; marco.vitolo@unimore.it (M.V.); marco.proietti@unimi.it (M.P.); S.Shantsila@liverpool.ac.uk (A.S.); 2 Cardiology Division, Department of Biomedical, Metabolic and Neural Sciences, University of Modena and Reggio Emilia, Policlinico di Modena, 41125 Modena, Italy; giuseppe.boriani@unimore.it; 3 Clinical and Experimental Medicine PhD Program, University of Modena and Reggio Emilia, 41125 Modena, Italy; 4 Department of Clinical Sciences and Community Health, University of Milan, 20138 Milan, Italy; 5 Geriatric Unit, IRCCS Istituti Clinici Scientifici Maugeri, 20138 Milan, Italy; 6 Aalborg Thrombosis Research Unit, Department of Clinical Medicine, Aalborg University, 9000 Aalborg, Denmark

**Keywords:** atrial fibrillation, cluster analysis, phenotype classification, stroke

## Abstract

Background and purpose: Given the great clinical heterogeneity of atrial fibrillation (AF) patients, conventional classification only based on disease subtype or arrhythmia patterns may not adequately characterize this population. We aimed to identify different groups of AF patients who shared common clinical phenotypes using cluster analysis and evaluate the association between identified clusters and clinical outcomes. Methods: We performed a hierarchical cluster analysis in AF patients from AMADEUS and BOREALIS trials. The primary outcome was a composite of stroke/thromboembolism (TE), cardiovascular (CV) death, myocardial infarction, and/or all-cause death. Individual components of the primary outcome and major bleeding were also assessed. Results: We included 3980 AF patients treated with the Vitamin-K Antagonist from the AMADEUS and BOREALIS studies. The analysis identified four clusters in which patients varied significantly among clinical characteristics. Cluster 1 was characterized by patients with low rates of CV risk factors and comorbidities; Cluster 2 was characterized by patients with a high burden of CV risk factors; Cluster 3 consisted of patients with a high burden of CV comorbidities; Cluster 4 was characterized by the highest rates of non-CV comorbidities. After a mean follow-up of 365 (standard deviation 187) days, Cluster 4 had the highest cumulative risk of outcomes. Compared with Cluster 1, Cluster 4 was independently associated with an increased risk for the composite outcome (hazard ratio (HR) 2.43, 95% confidence interval (CI) 1.70–3.46), all-cause death (HR 2.35, 95% CI 1.58–3.49) and major bleeding (HR 2.18, 95% CI 1.19–3.96). Conclusions: Cluster analysis identified four different clinically relevant phenotypes of AF patients that had unique clinical characteristics and different outcomes. Cluster analysis highlights the high degree of heterogeneity in patients with AF, suggesting the need for a phenotype-driven approach to comorbidities, which could provide a more holistic approach to management aimed to improve patients’ outcomes.

## 1. Introduction

Atrial fibrillation (AF) is the most common cardiac arrhythmia worldwide and despite substantial progress in its clinical management, it is still associated with high morbidity and mortality [1]. Of note, in most cases, AF occurs with other comorbidities and cardiovascular (CV) risk factors which confer a great clinical variety to this condition, implying the need for a holistic approach to the patients [2,3,4,5,6,7,8,9]. Given this heterogeneity, conventional classification only based on disease subtype or arrhythmia patterns may not adequately characterize the AF patient population. In recent years, several efforts have been made to improve the taxonomy of clinical classification of AF and other concomitant CV conditions (e.g., heart failure, valvular aortic stenosis, pulmonary arterial hypertension) by using different machine learning approaches [10,11,12]. One of the most popular unsupervised algorithms is cluster analysis which has recently been shown to be an excellent statistical approach for capturing relevant dissimilarities in disease phenotypes and identifying different patients’ clusters with distinct outcomes [13,14,15,16,17,18]. Cluster analysis is an exploratory statistical tool aimed at generating natural groups (or clusters) within the data [19]. The analysis is able to identify patients who are phenotypically homogenous and group them based only on measured different clinical characteristics between cases without investigators’ supervision [19]. Accordingly, by using pooled individual patient data from two randomized, open-label AF trials (AMADEUS and BOREALIS) [20,21], we aimed to identify different groups of AF patients who shared common clinical phenotypes applying cluster analysis and to evaluate the association between identified clusters and trial-adjudicated clinical outcomes.

## 2. Materials and Methods

### 2.1. Study Design and Cohort

For the present study, we used a pooled individual patient data of AMADEUS and BOREALIS randomized, open-label clinical trials [20,21]. The full details of the designs and the results of the two trials have been previously reported [20,21] and summarized in Appendix A. In brief, the AMADEUS trial [20] was a multicentre, randomized, open-label, assessor blind, non-inferiority study comparing the efficacy and safety of once-weekly subcutaneous idraparinux with adjusted-dose oral vitamin-k antagonists (VKA) in the prevention of thromboembolic events (TE) in patients with AF. The trial was stopped early because of excess clinically relevant bleedings in the idraparinux arm [20]. Comparably, the BOREALIS trial [21] was a multicentre, randomized, double-blind, double-dummy, non-inferiority study comparing the efficacy and safety of once-weekly subcutaneous idrabiotaparinux (or its placebo) with oral adjusted-dose warfarin in the prevention of stroke and systemic TE in patients with AF. The study was stopped prematurely by the sponsor for strategic/commercial, and not scientific, reasons [21]. Both trial protocols were approved by the institutional review board and all patients have provided written informed consent [20,21].

In our analysis, we included only AF patients treated with VKA. As required by cluster analysis, patients with missing data for any variables were excluded.

### 2.2. Study Outcomes

In this pooled analysis, we included all outcomes collected from the initiation of the treatment to the end of the studies. All outcomes were defined according to the original trials and have been previously reported [20,21]. Suspected outcome events in both trials were assessed by each independent central adjudication committee unaware of the treatment assignment. For the purpose of this analysis, the primary outcome was a composite of stroke/TE, CV death, myocardial infarction, and/or all-cause death. Individual components of the composite outcome and major bleeding (i.e., fatal bleeding, intracranial, or affected another critical anatomical site, or overt bleeding with a fall in the haemoglobin level of ≥20 g/L or requiring transfusion of ≥2 units of red blood cells). For the present analysis, we used only the first event of each outcome.

### 2.3. Statistical Analysis

We performed an agglomerative hierarchical cluster analysis for finding clusters of patients based on pre-specified clinical variables. The analysis aimed to identify the optimal number of clusters that were homogenous and indicative of a clinically relevant phenotypic subgroup of AF patients without prior knowledge of the outcomes. We used Ward’s minimum variance method in order to minimize the total within-cluster variance and we selected the squared Euclidean as a measure of distance or dissimilarity. The squared Euclidean distance was used because we selected only dichotomous variables. The following 12 baseline clinical variables were selected (all variables were considered as binary): sex, age > 75, anaemia (i.e., haemoglobin levels <120 g/L in women and <130 g/L in men), arterial hypertension, heart failure, diabetes mellitus, previous stroke/TE, coronary artery disease (CAD), chronic kidney disease (CKD) (i.e., eGFR< 60 mL/min), type of AF (permanent vs. paroxysmal/persistent), body mass index (BMI) classes (normal weight vs. overweight or obese) and use of any antiplatelets.

The algorithm begins with each element (i.e., patient) as a separate cluster and then proceeds with a “bottom-up” approach grouping each cluster with the most similar one until all clusters became one. The hierarchical clustering process is visually represented by a dendrogram graph in which vertical lines represent clusters that are joined together and the position of the line on the scale indicates the distance at which clusters were joined (the greater the difference in height, the more dissimilarity exists between clusters). The ideal number of clusters was not prespecified and was identified by examining the distances between cluster coefficients and further confirmed by visual inspection of the dendrogram created. By using this approach, we found that the grouping became more heterogeneous after being expanded to four clusters. Therefore, a 4-cluster model was used for this analysis. Once clusters were identified, we assessed the association between clusters and clinical characteristics and outcomes.

Continuous variables are reported as the median and interquartile range (IQR) or mean and standard deviation (SD). Categorical variables are described as counts with percentages. Among-clusters comparisons were conducted using the Kruskal-Wallis test for continuous and ordinal variables and using the Chi-square test or Fisher’s exact test (if any expected cell count was less than five) for categorical variables. Unadjusted and multivariate-adjusted Cox proportional hazards models were used to estimate the association between clusters and clinical outcomes. We built two multivariate models: for assessing all-cause death and the composite outcome of stroke/TE, CV death, myocardial infarction, and/or all-cause death, Model 1 was adjusted for CHA_2_DS_2_VASc score, and Model 2 was adjusted for age, sex, and type of AF. To evaluate the association between clusters and major bleeding events, Model 1 was adjusted for HASBLED score instead of the CHA_2_DS_2_VASc score whereas Model 2 was adjusted for the same covariates as reported above (i.e., age, sex, and type of AF). The hazard ratio (HR), the 95% confidence interval (CI), and the corresponding *p*-value were reported. Kaplan-Meier curves of outcomes were assessed and comparisons among different clusters were made using the Log-rank test. In all analyses, a two-tailed *p*-value of <0.05 was considered statistically significant. Statistical analysis was performed using SPSS^®^ (version 26).

## 3. Results

**Figure 1:**
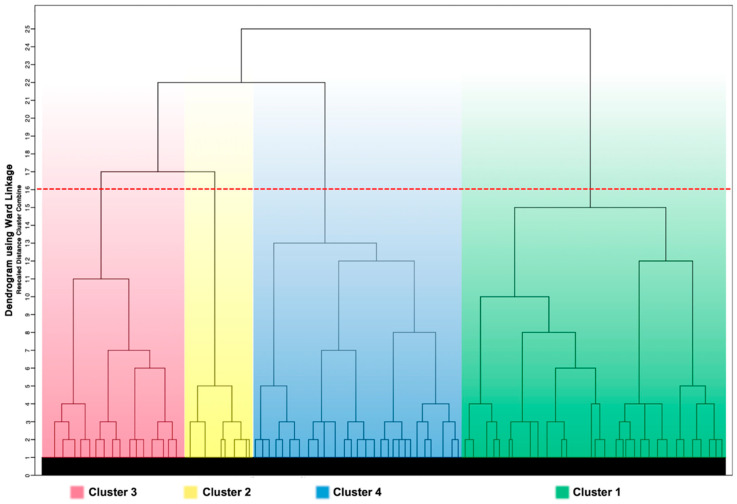
Dendrogram generated by hierarchical clustering process showing the four AF clusters. The dendrogram graph is the visual representation of the hierarchical clustering process. Vertical lines are clusters that are joined together and the position of the line on the scale indicates the distance at which clusters were joined (the greater the difference in height, the more dissimilarity exists between clusters). The red line indicates the stopping location.

Among the original 4169 AF patients treated with VKA from the merged dataset of the AMADEUS and BOREALIS studies, 3980 patients had complete baseline data for the 12 pre-specified clinical variables and were included in our study. The analysis identified four patient clusters as shown in the dendrogram (Figure 1). Baseline clinical characteristics according to the four clusters were compared and are reported in Table 1. The key characteristics of each patient cluster are described below.

### 3.1. Clusters

#### 3.1.1. Cluster 1 (n = 1530)

Cluster 1 was the largest cluster including 1530/3980 (38.4%) patients. Patients in this cluster were characterized by low rates of CV risk factors and comorbidities as reflected by the lowest CHA_2_DS_2_-VASc score compared to the other clusters (median 3, (2–4), *p* < 0.001). Particularly, patients were least likely to have hypertension and diabetes mellitus (both *p* < 0.001) and they had the lowest BMI (median 27 (24–30) kg/m^2^, *p* < 0.001).

#### 3.1.2. Cluster 2 (n = 397)

This was the smallest cluster and was characterized by patients being more likely to be male (60.7%) with a high burden of CV risk factors including the highest rates of diabetes mellitus (97.5%) and obesity (58.4%) (both *p* < 0.001). Patients in this cluster had the lowest rate of CAD (19.4%) and heart failure (14.9%) (both *p* < 0.001). Given the lowest rate of CAD, patients were the least likely to be treated with concomitant antiplatelets (1.3%, *p* < 0.001).

#### 3.1.3. Cluster 3 (n = 830)

Patients in this cluster were the youngest (median 65 (59–70) years) and the most likely to be male (66.4%). The key and distinguishing characteristics of this cluster consisted in the high burden of CV comorbidities and risk factors such as heart failure (77.7%), CAD (48.3%), hypertension (97.1%), and obesity (median BMI 30 (27–35)). Reflecting the high rate of CAD, the use of concomitant antiplatelet agents was higher compared to other clusters (53.3%, *p* < 0.001). Concerning AF patterns, Cluster 3 patients had the highest rate of permanent AF (62.8%, *p* < 0.001).

#### 3.1.4. Cluster 4 (n = 1221)

Cluster 4 was the second largest cluster (1223/3980, 30.7%). It was characterized by older patients (median 76, (70–79) years) who were more likely to be female compared to the other clusters (41.3%, *p* < 0.001). Cluster 4 had the unique characteristic of the highest rates of non-CV comorbidities, including anaemia (33.8%), CKD (35.6%), and previous TE (35.9%) (all *p* < 0.001 compared to other clusters). Given the high burden of comorbidities, patients in this Cluster had the highest CHA_2_DS_2_-VASc and HASBLED scores (median 5, (4–6) and median 2 (2–3), respectively, *p* < 0.001).

### 3.2. Associations with Clinical Outcomes

Table 2 compares the crude rates of major adverse events among Clusters. Over a mean follow-up of 365 (standard deviation (SD) 187) days, the occurrence of the composite outcome and all-cause death, was higher in Cluster 4 (8.3% and 6.6%) compared to Cluster 1 (3.1% and 2.5%), Cluster 2 (5.0% and 3.5%), and Cluster 3 (3.7% and 3.3%, respectively) (all *p* < 0.001). These findings were consistent also when considering individual components of the composite outcome and major bleeding events (Table 2).

**Figure 2:**
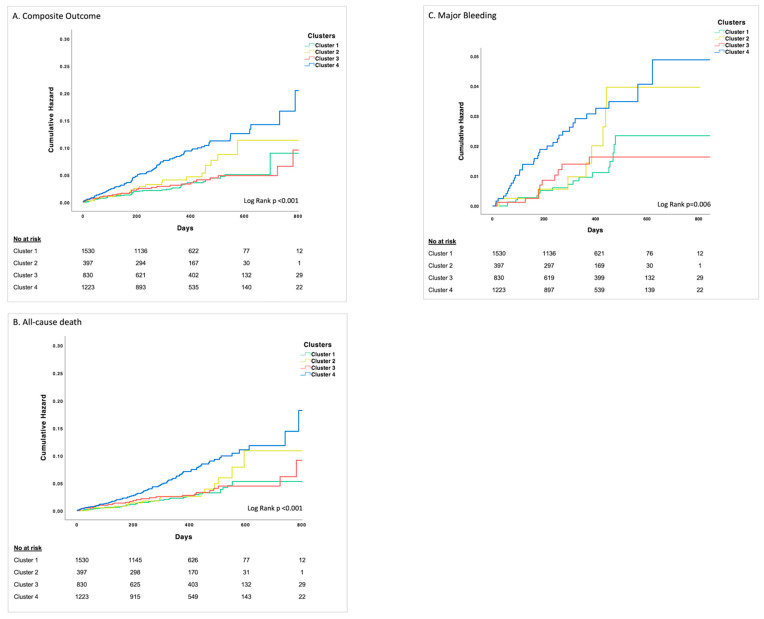
Kaplan-Meier curves for the composite outcome of stroke/thromboembolism, cardiovascular death, myocardial infarction and/or all cause-death (**A**), all-cause death (**B**), and major bleeding (**C**).

As shown in Kaplan Meier curves patients in Cluster 4 had the highest cumulative risk of outcomes (Figure 2A–C).

The unadjusted and multivariate-adjusted Cox proportional hazards analyses estimating the association between clusters and clinical outcomes are reported in Table 3. Cluster 1 was used as the comparator group since it was the Cluster with lower rates of outcomes compared to other Clusters. In the unadjusted Cox analysis, when compared with Cluster 1, Cluster 4 had a significantly higher risk for the composite outcome (HR 2.59, 95% CI 1.83–3.66), all-cause death (HR 2.47, 95% CI 1.68–3.63), and major bleeding (HR 2.42, 95% CI 1.35–4.34). After the adjustments according to multivariate models (Model 1 (M1)) adjusted for CHA_2_DS_2_-VASc score for the composite outcome and all-cause death or HASBLED for major bleeding outcome, Model 2 (M2) adjusted for age, sex, and type of AF) Cluster 4 was independently associated with an increased risk for the composite outcome (M1: HR 1.86, 95% CI 1.26–2.73; M2: HR 2.43, 95% CI 1.70–3.46), all-cause death (M1: HR 1.82, 95% CI 1.18–2.81; M2: HR 2.35, 95% CI 1.58–3.49), and major bleeding (M1: HR 1.96, 95% CI 1.07–3.57; M2: HR 2.18, 95% CI 1.19–3.96), with no significant association between Cluster 2 and Cluster 3 with the risk of all outcomes (Table 3).

## 4. Discussion

To the best of our knowledge, the present study is one of the largest analyses of AF patients based on this statistical approach for trial-adjudicated clinical outcomes. The main findings of our study are as follows: (i) Cluster analysis was able to identify four statistically-driven groups of AF patients which are clinically relevant and present distinct phenotype characteristics; (ii) each patient Cluster was markedly different from the other not only on the basis of measures which commonly drive conventional classifications of AF (e.g., AF patterns) but each Cluster significantly varied among the measures of demographic characteristics, CV-risk factors, comorbidities, and concomitant treatment; (iii) importantly, the identified Clusters exhibited different risk for all-cause death and major clinical adverse events.

Conventional classifications, only based on AF patterns (i.e., paroxysmal, persistent, and permanent) might be limited by the natural progression of the disease and often overlook the variety of underlying conditions that are commonly associated with AF [22]. In this study, we have applied a novel statistical approach to a large cohort of anticoagulated AF patients derived from two multicentre randomized clinical trials. Our results underline the great heterogeneity of AF highlighting the need for a disease classification of AF patients that should encompass all possible clinical phenotypes.

This cluster analysis reinforces the importance of identifying possible patient phenotypes which share clinical characteristics and common risk factors. Despite cluster analysis might be not easily applied in daily clinical practice, the information provided by our study further strengthen the concept that AF patients need improved clinical characterization beyond the mere classification by the arrhythmia patterns

Our analysis found four main clinical phenotypes. The first cluster was characterized by low rates of CV risk factors and comorbidities, such as hypertension, diabetes mellitus, and the lowest rate of permanent AF. Not surprisingly, this cluster had the lowest rates of adverse outcomes including all-cause death, CV death, and major bleeding. Cluster 2 and Cluster 3, were characterized by a high burden of CV risk factors and CV comorbidities, as reflected by a higher CHA_2_DS_2_-VASc score. Despite these two clusters seeming similar, the key distinguishing characteristics of Cluster 3 compared to Cluster 2 is the higher prevalence of cardiac comorbidities, such as heart failure or CAD which are significantly lower in the latter. Finally, Cluster 4 is characterized by older patients with the unique characteristic of the highest rates of non-CV comorbidities including anaemia, CKD, and previous TE thus conferring a higher risk of adverse outcomes during the follow-up despite oral anticoagulation.

Cluster analysis has been recently applied in other observational AF cohorts. Indeed, if we review the available data in the literature, some important comparisons can be made between our study and previous analyses based on this statistical approach. One of the first studies that applied cluster analysis in a large cohort of AF patients was conducted by Inohara et al. [10]. Using data from the ORBIT-AF Registry, the study showed that unsupervised cluster analysis was able to identify four clinically relevant AF phenotypes (i.e., low comorbidity cluster, young/behavioural disorder cluster, device implantation cluster, atherosclerotic-comorbid cluster) [10]. Similar to our analysis, when compared to the low comorbidity cluster, the occurrence of major adverse CV events was significantly higher in the three other clusters, even after the adjustment with CHA_2_DS_2_VASc score (younger/behavioural disorder cluster: HR, 1.49; 95% CI, 1.10–2.00; device implantation cluster: HR, 1.39; 95% CI, 1.15–1.68; and the atherosclerotic comorbid cluster: HR, 1.59; 95% CI, 1.31–1.92) [10]. More recently, a cluster analysis based on Japanese AF patients from the cohort of the Keio Interhospital Cardiovascular Studies for AF (KiCS-AF) registry [11] similarly detected an atherosclerotic comorbid AF cluster phenotype, as was found in the US cohort from the ORBIT-AF registry [10,11]. However, conventional classifications, such as type of AF or LA size, did not drive cluster formation in the US cohort [10] while contributing to the formation of clusters in the KiCS-AF registry [11]. Moreover, in the analysis from the ORBIT-AF registry [10], one of the clusters identified was characterized by younger age, high rates of liver disease, alcohol or drug abuse, and current smoking (namely, the younger//behavioural cluster). Interestingly, these key variables that significantly contributed to the cluster formation in the ORBIT-AF cohort, were lower, if not completely absent, in the Japanese cohort [10,11].

These findings further emphasize that determinants of AF may greatly vary across different regions and enrich the debate on the great heterogeneity existing among AF patients worldwide [23]. Hence, the move towards a more holistic and integrated approach to AF management, as promoted in recent AF guidelines [24,25,26]. With the introduction of the ABC (Atrial fibrillation Better Care) pathway, i.e., ’A’ Anticoagulation/Avoid stroke; ‘B’ Better symptom management; ‘C’ Cardiovascular and Comorbidity optimization [24], identifying clinically relevant phenotype groups of AF patients could be useful for a more tailored clinical approach. From the perspective of such an integrated approach, cluster analysis may be a good ally to further understand the complex nature of AF patients, facilitate management decision-making, and improve their outcomes. For example, patients in Cluster 2 had a high burden of CV risk factors but low rates, or not as of yet phenotypically expressed, CV comorbidities such as CAD or heart failure. Thus, for these patients, lifestyle modifications and strict control of CV factors could be representing the most effective treatments which should be promptly undertaken to prevent adverse outcomes. In contrast, Cluster 4, which is characterized by a higher burden of non-CV comorbidities, showed a significantly higher risk for all-cause death and CV events despite the use of oral anticoagulation, highlighting that oral anticoagulation alone without an optimal treatment of comorbidities, is insufficient to improve clinical outcomes.

In line with this concept, especially in high-risk patients, cluster analysis re-emphasizes the importance of more comprehensive management of AF patients which should encompass all three steps of the ABC pathway [27,28,29].

Cluster analysis is not to be considered as a formal proposal of a new classification system for AF. Our aim was to apply a novel validated statistical approach to a large cohort of AF patients that can capture relevant clinical factors and phenotypic similarities often overlooked in conventional classifications. Our study, therefore, suggests that along with conventional classifications, new strategies that are able to be clinically relevant in defining phenotype groups could be a step toward precision medicine and a more comprehensive characterisation of AF patients.

### Limitations

Some limitations of our study should be acknowledged. First, identified clusters may vary according to patient characteristics and available data. We arbitrarily selected the most common comorbidities and risk factors that characterize AF patients. We are conscious that the incorporation of more clinical characteristics, such as cognitive impairment that has been demonstrated to have a relevant impact on AF patient outcomes [8,9,30], might yield different results. However, since cluster analysis necessitates complete data on individual patients, we chose to select a relatively small number of well-documented variables in order to include as many patients as possible.

Second, the optimal number of clusters may be difficult to determine since different statistical algorithms may generate different results and the final selection of four clusters was based in part on investigator discretion. Despite the strength of this analysis as the adjudicated clinical outcomes from the posthoc analysis of the two trial cohorts, the multivariate Cox regression analysis may be limited due to the relatively small number of events. As this study is a post hoc ancillary analysis of two controlled clinical trials in which patients were treated only with VKA, the population might not fully reflect a contemporary real-world AF population. Treatment strategies and clinical practice have changed over time, partly limiting the generalization of the results. Nevertheless, we considered a large cohort of AF patients with a high level of data quality and with centrally adjudicated clinical outcomes. For these reasons, our analysis should be considered to be hypothesis-generating and further studies are warranted to confirm our findings and the strength of this statistical approach.

## 5. Conclusions

Among a large cohort of AF patients from the AMADEUS and BOREALIS trials, cluster analysis identified four different clinically relevant phenotypes that had unique clinical characteristics and different outcomes. Cluster analysis highlights the high degree of heterogeneity in patients with AF suggesting the need for a phenotype-driven approach to comorbidities which could provide a more holistic approach to management aimed to improve patients’ outcomes.

## Figures and Tables

**Table 1:** Baseline characteristics of patients stratified by clusters.

	Cluster 1 N = 1530	Cluster 2 N = 397	Cluster 3 N = 830	Cluster 4 N = 1223	*p* Value
Age, years, median (IQR)	71 (64–76)	68 (61–73)	65 (59–70)	76 (70–79)	<0.001
Age, years, mean (SD)	69.4 (9.4)	67.5 (8.0)	64.4 (7.9)	74.4 (8.0)	<0.001
Females, n (%)	516 (33.7)	160 (40.3)	279 (33.6)	505 (41.3)	<0.001
BMI, median (IQR)	27 (24–30)	31 (27–35)	30 (27–35)	28 (25–31)	<0.001
BMI Classes, n (%)					<0.001
*Normal Weight*	474 (31.0)	42 (10.6)	22 (2.7)	292 (23.9)	
*Overweight*	636 (41.6)	123 (31.0)	324 (39.0)	527 (43.1)	
*Obese*	420 (27.4)	232 (58.4)	484 (58.3)	404 (33.0)	
Permanent AF, n (%)	779 (50.9)	242 (61.0)	521 (62.8)	664 (54.3)	<0.001
CHA_2_DS_2_-VASc, median (IQR)	3 (2–4)	4 (3–5)	4 (3–5)	5 (4–6)	<0.001
HAS-BLED, median (IQR)	2 (1–2)	2 (1–2)	2 (1–2)	2 (2–3)	<0.001
Previous TE, n (%)	466 (30.5)	51 (12.8)	116 (14.0)	439 (35.9)	<0.001
Hypertension, n (%)	1041 (68.0)	366 (92.2)	806 (97.1)	1144 (93.5)	<0.001
Heart Failure, n (%)	273 (17.8)	59 (14.9)	645 (77.7)	572 (46.8)	<0.001
Diabetes Mellitus, n (%)	62 (4.1)	387 (97.5)	275 (33.1)	307 (25.1)	<0.001
CAD, n (%)	362 (23.7)	77 (19.4)	401 (48.3)	628 (51.3)	<0.001
CKD, n (%)	61 (4.0)	37 (9.3)	24 (2.9)	435 (35.6)	<0.001
Anaemia, n (%)	43 (2.8)	31 (7.8)	0 (0)	413 (33.8)	<0.001
Any Antiplatelet Drugs, n (%)	121 (7.9)	5 (1.3)	442 (53.3)	404 (33.0)	<0.001
TTR, median (IQR)	60 (46—71)	57 (43–73)	58 (40–73)	59 (43–73)	0.10

AF = atrial fibrillation; BMI = body mass index; CAD = coronary artery disease; CKD = chronic kidney disease; IQR = interquartile range; N = number; TE = thromboembolic events; TTR = time in therapeutic range.

**Table 2:** Major adverse events stratified by clusters.

	Cluster 1 N = 1530	Cluster 2 N = 397	Cluster 3 N = 830	Cluster 4 N = 1223	*p* Value
Composite outcome, n (%)	47 (3.1)	20 (5.0)	31 (3.7)	102 (8.3)	<0.001
All-cause death, n (%)	38 (2.5)	14 (3.5)	27 (3.3)	81 (6.6)	<0.001
Stroke/TE, n (%)	15 (1.0)	8 (2.0)	10 (1.2)	30 (2.5)	0.013
Cardiovascular death, n (%)	17 (1.1)	5 (1.3)	13 (1.6)	41 (3.4)	<0.001
Myocardial infarction, n (%)	2 (0.1)	2 (0.5)	3 (0.4)	10 (0.8)	0.05
Major bleeding, n (%)	17 (1.1)	8 (2.0)	10 (1.2)	34 (2.8)	0.005

N, number; SE, systemic thromboembolism; composite outcome = stroke/TE, CV death, myocardial infarction, and/or all-cause death.

**Table 3:** Unadjusted and adjusted Cox regression analysis for all-cause death and the composite outcome.

	Unadjusted Analysis			Multivariate Analysis [Model 1]			Multivariate Analysis [Model 2]		
	HR	95% CI	*p*	HR	95% CI	*p*	HR	95% CI	*p*
Composite outcome *									
Cluster 1 (ref)	-	-	-	-	-	-	-	-	-
Cluster 2	1.62	0.96–2.73	0.07	1.48	0.88–2.50	0.14	1.63	0.96–2.75	0.07
Cluster 3	1.07	0.68–1.69	0.77	0.94	0.59–1.49	0.81	1.12	0.70–1.79	0.63
Cluster 4	2.59	1.83–3.66	<0.001	1.86	1.26–2.73	0.002	2.43	1.70–3.46	<0.001
All cause-death *									
Cluster 1 (ref)	-	-	-	-	-	-	-	-	-
Cluster 2	1.38	0.75–2.55	0.30	1.27	0.66–2.36	0.44	1.37	0.74–2.53	0.31
Cluster 3	1.12	0.68–1.85	0.64	1.01	0.61–1.66	0.98	1.16	0.69–1.92	0.58
Cluster 4	2.47	1.68–3.63	<0.001	1.82	1.18–2.81	0.006	2.35	1.58–3.49	<0.001
**Major Bleeding §**									
Cluster 1 (ref)	-	-	-	-	-	-	-	-	-
Cluster 2	1.76	0.76–4.09	0.18	1.89	0.81–4.39	0.13	1.90	0.81–4.44	0.13
Cluster 3	1.01	0.46–2.20	0.98	1.01	0.46–2.21	0.97	1.21	0.54–2.71	0.63
Cluster 4	2.42	1.35–4.34	0.003	1.96	1.07–3.57	0.02	2.18	1.19–3.96	0.01

CI = confidence interval; HR = hazard ratio; ref = reference. * Model 1: adjusted analysis for CHA_2_DS_2_VASc score; Model 2: adjusted analysis for age, sex, and type of atrial fibrillation; ^§^ Model 1: adjusted analysis for HASBLED; Model 2: adjusted for age, sex, and type of atrial fibrillation.

## Data Availability

All supporting data are available within the article.

## Appendix A. Supplemental Methods

The AMADEUS trial (20) was a multicentre, randomized, open-label non-inferiority study with blinded assessment of outcome that compared fixed-dose idraparinux with conventional anticoagulation by dose-adjusted oral vitamin K antagonist (VKA) therapy for the prevention of thromboembolism in atrial fibrillation (AF) patients. Inclusion criteria: ECG-documented non-valvular AF and an indication for long-term anticoagulation, based on the presence of at least 1 of the following risk factors: previous ischemic stroke, transient ischemic attack or systemic embolism, hypertension requiring drug treatment, left ventricular dysfunction (left ventricular ejection fraction <45% or symptomatic congestive heart failure), age > 75 years, or age 65 to 75 years with either diabetes mellitus or symptomatic coronary artery disease (previous myocardial infarction or angina pectoris). Exclusion criteria included: indication for VKA other than AF, active bleeding or high risk of bleeding, severe renal failure (creatinine clearance <10 mL/min) and/or severe hepatic disease, uncontrolled hypertension.

The BOREALIS trial (21) was a multicentre, randomized, double-blind, double-dummy, non-inferiority trial comparing idrabiotaparinux (or its placebo) and dose-adjusted warfarin (or its placebo) for the prevention of thromboembolism in patients with AF. Inclusion criteria were non-valvular, ECG- documented AF with an indication for long-term VKA therapy based on the presence of previous ischemic stroke, transient ischemic attack, or systematic thromboembolism and/or at least two of the following risk factors: hypertension requiring drug treatment; moderately or severely impaired left ventricular function and/or heart failure; age ≥ 75 years; or diabetes mellitus. Exclusion criteria included: severe renal failure (creatinine clearance < 30 mL/min), indication for VKA other than AF, stroke or TIA within 5 days, uncontrolled hypertension, history of intracranial, intraocular, spinal, overt gastrointestinal, retroperitoneal, or traumatic intra- articular bleeding or life-threatening bleeding; active bleeding or high risk of bleeding.
